# Micromotion of Dental Implants: Basic Mechanical Considerations

**DOI:** 10.1155/2013/265412

**Published:** 2012-11-20

**Authors:** Werner Winter, Daniel Klein, Matthias Karl

**Affiliations:** ^1^Department of Mechanical Engineering, University of Erlangen-Nuremberg, Egerlandstraße 5, 91058 Erlangen, Germany; ^2^Department of Prosthodontics, University of Erlangen-Nuremberg, Glueckstraße 11, 91054 Erlangen, Germany

## Abstract

Micromotion of dental implants may interfere with the process of osseointegration. Using three different types of virtual biomechanical models, varying contact types between implant and bone were simulated, and implant deformation, bone deformation, and stress at the implant-bone interface were recorded under an axial load of 200 N, which reflects a common biting force. Without friction between implant and bone, a symmetric loading situation of the bone with maximum loading and displacement at the apex of the implant was recorded. The addition of threads led to a decrease in loading and displacement at the apical part, but loading and displacement were also observed at the vertical walls of the implants. Introducing friction between implant and bone decreased global displacement. In a force fit situation, load transfer predominantly occurred in the cervical area of the implant. For freshly inserted implants, micromotion was constant along the vertical walls of the implant, whereas, for osseointegrated implants, the distribution of micromotion depended on the location. In the cervical aspect some minor micromotion in the range of 0.75 *μ*m could be found, while at the most apical part almost no relative displacement between implant and bone occurred.

## 1. Introduction

**Figure 1:**
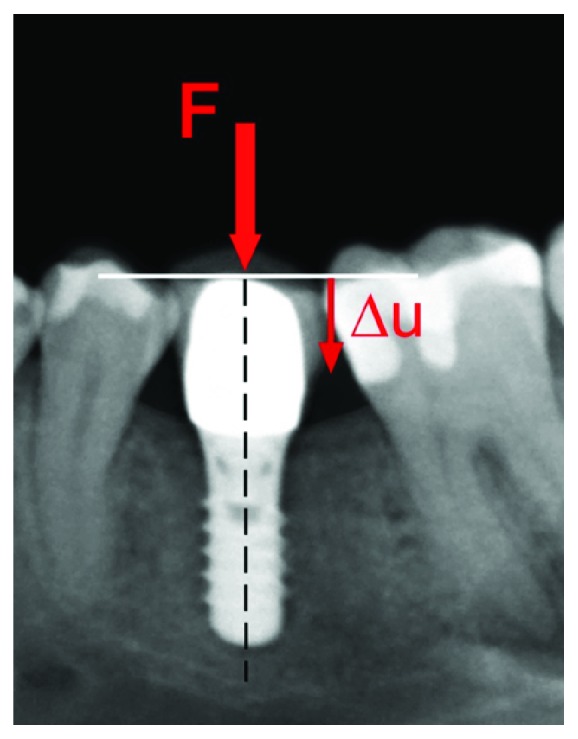
Single tooth implant used for replacing the first molar in the lower left mandible. An axial force acting on the occlusal surface of the restorations may displace the implant relative to the surrounding bone.

Micromotion of dental implants has been defined as minimal displacement of an implant body relative to the surrounding tissue which cannot be recognized with the naked eye [1] (Figure 1). Various authors have shown that excessive micromotion may interfere with the process of osseointegration of dental implants [2, 3]. Although exact data are missing, it has been postulated that micromotion between implant and bone must not surpass a threshold value of 150 micrometer (*μ*m) for successful implant healing [4–6].

In traditional loading protocols, where implants are allowed to heal undisturbed for periods of several months, the issue of implant micromotion is of limited importance. With the advent of modern treatment concepts including early and immediate loading of dental implants [7, 8], with implants being restored early in the healing phase, the issue of implant micromotion has gained significant importance [4, 5].

Numerous reports trying to relate clinical parameters to the phenomenon of implant micromotion can be found in the dental literature [8–11]. The nonuniform nomenclature, the varying experimental settings, and the partially contradicting results presented on the one hand indicate the complexity of the topic but on the other hand emphasize the need for clarifying basic engineering principles.

From a biomechanical perspective, successful osseointegration of dental implants depends on the way mechanical stresses and strains are transferred to the surrounding bone and tissues. The multiple factors hereby affecting stress and strain transfer include the type of loading that occurs, the type of implant-bone interface being present, the length and diameter of the implant, implant geometry and its surface texture, and the quality and quantity of the surrounding bone [13–19]. Only by understanding the most critical of these variables, strategies for optimizing implant stabilization can be developed. For determining how implant mobility, often referred to as micromotion, relative motion, micromovement, and so forth, or implant loading affects bone response, a closer look at implant deformation, bone deformation, and stress or strain at the implant-bone interface is required [20].

In this context, it was the purpose of this paper to mechanically describe the phenomenon of micromotion occurring between implant and alveolar bone using simple spring models, continuum mechanics models, and 3D-Finite-Element models simulating varying contact types between implant and bone [21–23].

## 2. Material and Methods

### 2.1. Basic Considerations

**Figure 2:**
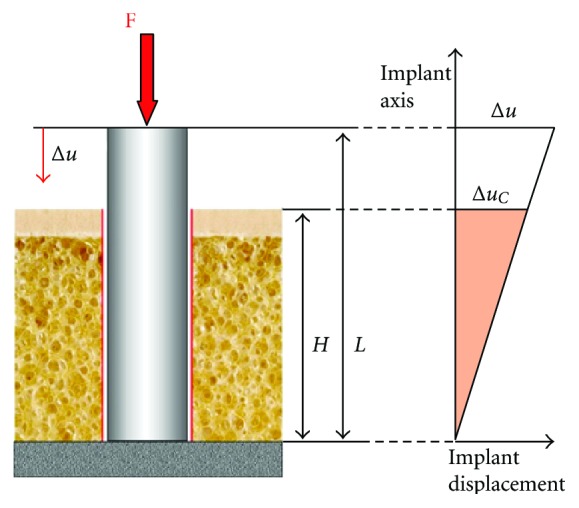
Description of scenario 1 with a dental implant resting on a fixed apical surface, with no contact existing between the vertical implant walls and the walls of the bony socket (left). When the implant is loaded vertically, deformation of the implant occurs mainly in the coronal part and decreases towards the apex. Similarly, relative displacement between implant and bone diminishes towards the apex (right).

Three basic scenarios reflecting different anchoring situations of dental implants were considered. In scenario 1, the implant rests on an apically located fixed surface but neither has contact to cortical bone nor to trabecular bone at the vertical walls of the implant (Figure 2). In this situation, maximum implant deformation under vertical loading occurs in the coronal part and diminishes gradually towards the apex. As a result, micromotion between the implant and the vertical walls of the socket also decreases towards the apical part of the implant. Axial deformation of the implant as a consequence of vertical loading can be calculated according to

(1) $$\begin{array}{c} \Delta u=\frac{F}{EA/L}=\frac{F}{c} \end{array}$$

with *F* standing for the vertical force applied, *E* being the Young's modulus of the implant, *A* being the cross section of the implant, *L* being the length of the implant, and *c* being the stiffness of the implant. Maximum micromotion Δ*u*_*c*_  at the cortical area can then be calculated according to

(2) $$\begin{array}{c} \Delta u_{c}=\frac{\Delta u}{L}H \end{array}$$

with *H* reflecting the height of cortical and trabecular bone around the implant.

**Figure 3:**
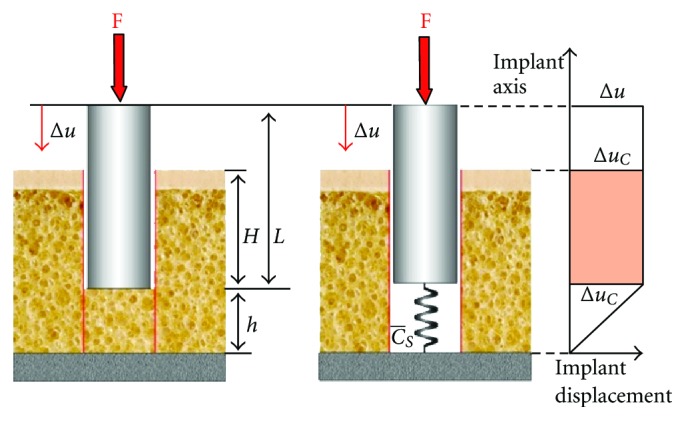
Description of scenario 2, where the implant rests on a layer of elastic trabecular bone with no contact existing between the vertical implant walls and the walls of the bony socket (left). The apically located layer of bone may be substituted by a spring which is compressed when an axial load is applied on the implant (center). Due to the great difference in elastic modulus between implant and trabecular bone, relative implant displacement is independent from the region of the implant considered (right).

For scenario 2, the fixed apical rest of the implant was altered by adding a layer of elastic trabecular bone apically to the implant. Here, an axial force acting on the implant predominantly causes compression of the elastic material the implant is resting on. Due to the drastically smaller elastic modulus of trabecular bone as compared to titanium, the deformation of the implant can be neglected and the relative movement between implant and bone is independent from the region of the implant considered (Figure 3). In this situation, implant displacement may be calculated according to

(3) $$\begin{array}{c} \Delta u=\frac{F}{E_{s}A/h}\left[1+\frac{L}{h}\frac{E_{s}}{E}\right]=\frac{F}{\overset{-}{C_{s}}}\left[1+\beta \right], \end{array}$$

where *E*_*s*_ is the Young's modulus of the trabecular bone and *h* the height of the bone underneath the implant. 

Taking into account that the Young's modulus of the implant is much greater than the Young's modulus of the trabecular bone (*E* ≫ *E*_*s*_)  it is accepted *β* ≪ 1  and furthermore the approximation for the micromotion

(4) $$\begin{array}{c} {\Delta u}_{c}=\Delta u\approx \frac{F}{\overset{-}{C_{s}}}. \end{array}$$

Consequently, micromotion at the cortical area of the implant and the relative micromotion between implant and bone are identical, both being related to the stiffness $\overset{-}{C_{s}}$ of the trabecular bone underneath the implant.

**Figure 4:**
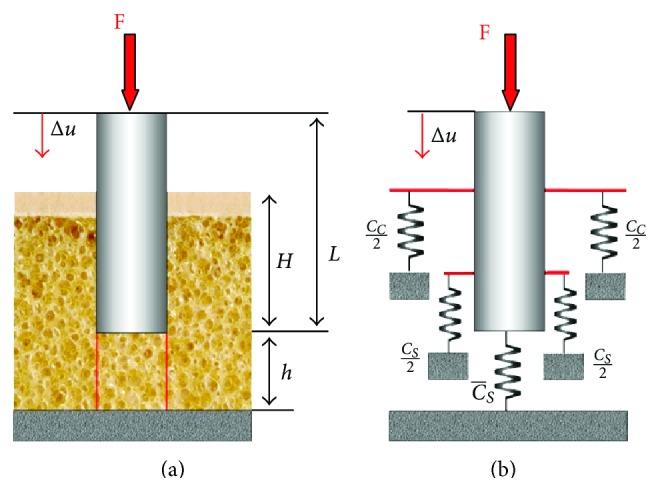
Scenario 3 showing an implant elastically supported by cortical and trabecular bone (a). The elastic support in the different regions can be replaced by a system of springs (b).

Further approximating the clinical situation of an osseointegrated implant, in scenario 3 the implant is elastically supported by surrounding cortical and trabecular bone. Due to the fixed contact between implant and bone, micromotion at this interface does not occur, when the implant is axially loaded (Figure 4). Neglecting the deformation of the implant (*E* ≫ *E*_*s*_) the implant displacement may be calculated according to

(5) $$\begin{array}{c} \Delta u=\frac{F}{\overset{-}{C_{s}}}\frac{1}{\left(1+\left(C_{s}/\overset{-}{C_{s}}\right)+\left(C_{c}/\overset{-}{C_{s}}\right)\right)}. \end{array}$$

Under the circumstances of scenario 3, no relative micromotion between implant and bone exists. The displacement of the implant equals the micromotion depending on the stiffness of both cortical and trabecular bone surrounding the implant.

**Figure 5:**
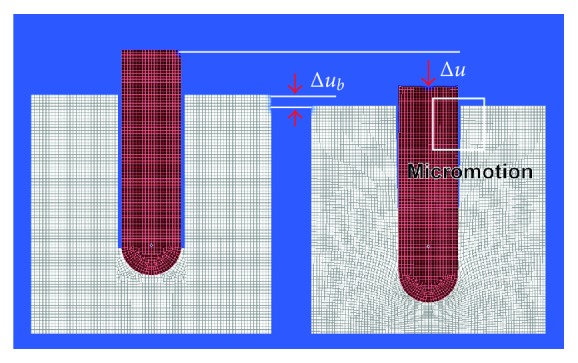
Without contact between implant and bone, an axial force acting on the implant causes implant dislocation as a result of elastic deformation of bone predominantly in the periapical region of the implant. Left: unloaded implant; right: loaded implant with implant displacement Δ*u* and displacement of cortical bone Δ*u*_*b*_ (displacement of a reference mark on bone).

**Figure 6:**
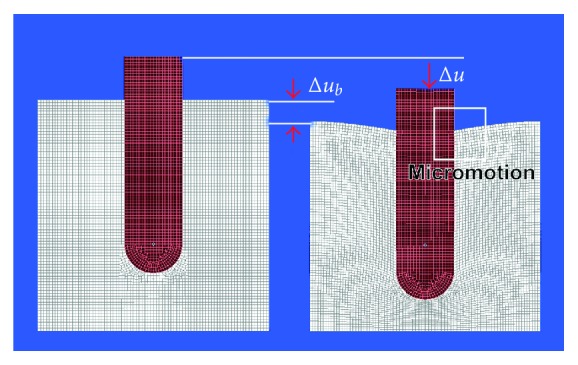
Considering an osseointegrated implant with contact between the implant surfaces and bone, axial implant loading causes elastic deformation of bone in all areas but no relative displacement between implant and bony socket, that is, no micromotion, occurs. Left: unloaded implant; right: loaded implant with implant displacement Δ*u* and displacement of cortical bone Δ*u*_*b*_ (displacement of a reference mark on bone).

Further approximating clinical reality, continuum mechanics models were considered revealing implant displacement due to elastic deformation of bone when no contact between implant and bone was modelled in a plane strain FE-model (Figure 5). This model correlates with scenario 2 described above (Figure 3). Simulating contact between implant and bone, bone is also elastically deformed in the cervical portion of the implant when an axial load is exerted; however, no relative micromotion between implant and bone occurs at the interface (Figure 6), and the implant displacement (micromotion) is related to the elastic properties of the cortical and trabecular bone.

### 2.2. Finite Element Analysis

**Figure 7:**
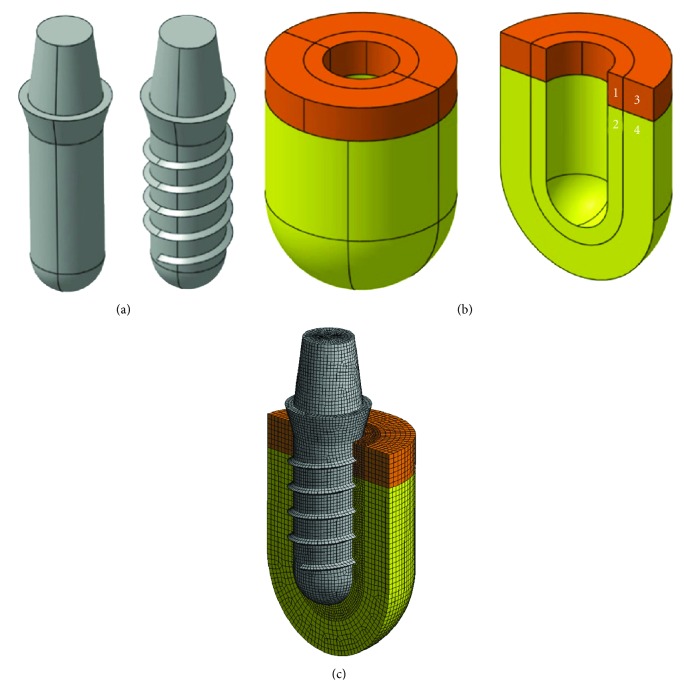
(a) Three-dimensional finite element models of dental implants with and without threads [12]. (b) Three-dimensional finite element model of a bony implant socket with cortical and trabecular bone. Areas (1) and (2) surrounding the implant are designed as an intermediate layer allowing the elastic modulus to be set independently from areas (3) and (4) representing native bone which is not affected by healing processes occurring during osseointegration [12]. (c) Three-dimensional finite element model of a single implant embedded in a bone segment consisting of cortical and trabecular bone (calculations were done on a complete model; for illustration purposes the model is cut in half) [12].

For a more realistic representation of clinical conditions, three-dimensional FE models [12] of dental implants with and without threads were generated (Figure 7(a)) which were subsequently embedded in a bony socket consisting of cortical and trabecular bone and an intermediate layer surrounding the implant (Figure 7(b)). The geometry of the models was generated with a CAD Program (SolidWorks 2011, SolidWorks Deutschland GmbH, Haar, Germany) and imported in a FE program (ANSYS Workbench 12, ANSYS Inc., Canonsburg, PA, USA).

Combining both components, three-dimensional FE models (Figure 7(c)) were obtained for evaluating micromotion between implant and bone when an axial vertical force of 200 N was exerted which reflects an average biting force [24, 25]. Different stages of osseointegration were simulated by altering the elastic modulus of the intermediate bone layer [21–23]. The contact type between implant and bone could be modified as friction free, only transferring compressive forces and allowing for sliding and gap formation, to friction (friction coefficient 0.3) and force fit, respectively [21].

In general, isotropic linear model parameters were applied, defining the contact type between the different layers of bone as “bond.” Out of the large number of possible solutions for solving contact problems, the augmented Lagrange method was chosen as accompanying optimization method. This method was applied for defining all contacts not allowing contacting components to penetrate each other. Poisson's ratio was set at 0.3 for all materials. Based on the results of previous investigations [22, 23] indicating that the size of the models was sufficient for evaluating micromotion, model dimensions were reduced to a minimum and the borders of the models were fixed. Depending on model type, 160000 hexaeder elements and 600000 to 650000 nodes were used to set up the models using the elastic modules given in Table 1. Based on the fact that the elastic values and the strength limits of biologic materials *in vivo*—such as the bone-implant interface—are highly complex [24], only two states of osseointegration were considered (starting point and end point of osseointegration). These different states were modelled by different elastic values in the areas (1) and (2) in Figure 7(b).

Results of all simulations were recorded as von Mises equivalent stress in addition to contour plots of global displacement. 

**Figure 8:**
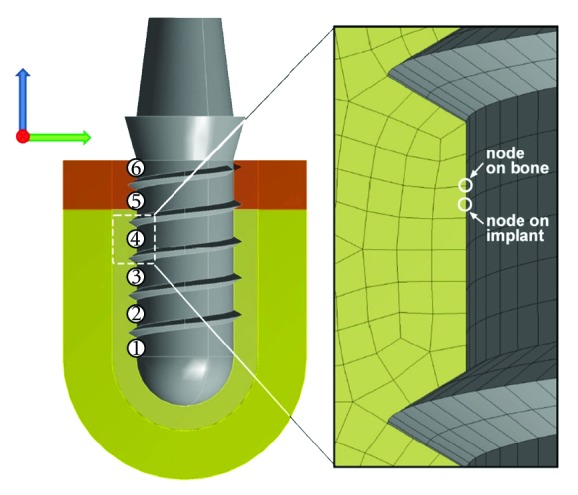
Definition of micromotion at the implant bone interface. Six corresponding nodes on the implant and on the bone were used as reference marks. For determining the relative displacement of two corresponding nodes on bone and implant, the displacement of a specific reference mark on the bone was subtracted from the displacement of the corresponding reference mark on the implant.

For calculating relative displacement between implant and bone (relative micromotion), a total of six corresponding nodes at the implant bone interface were established as reference marks. As the displacement of a specific reference mark on the implant represents both displacement of bone and implant, the displacement of the corresponding reference mark on the bone (Figure 8) was subtracted.

## 3. Results

**Figure 9:**
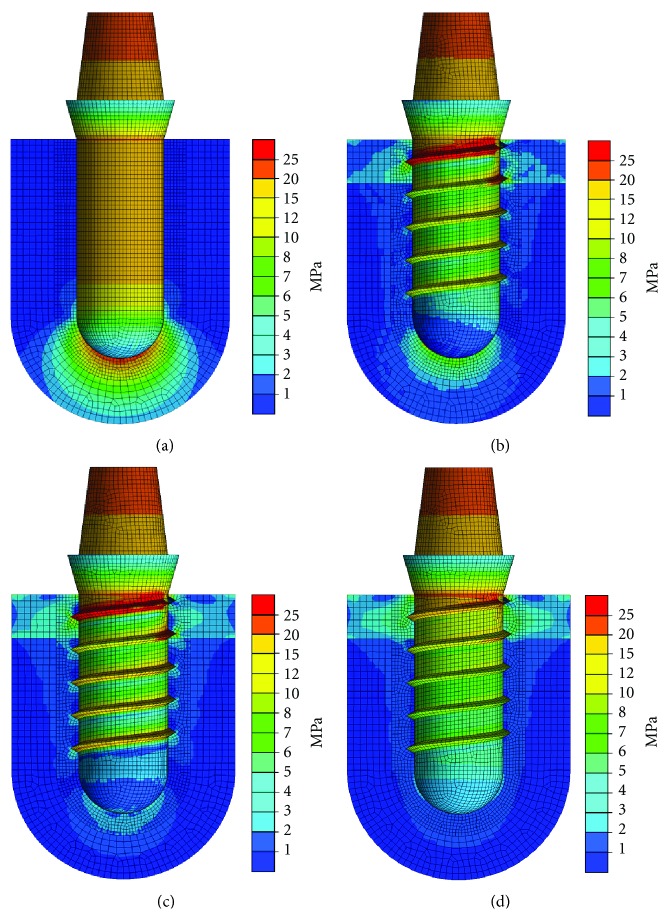
Distribution of von Mises equivalent stress around implants loaded with 200 N axial vertical force [12]: cylindrical implant without friction between implant and bone (a), threaded implant without friction between implant and bone (b), threaded implant with friction between implant and bone (coefficient of friction: 0.3) (c), and threaded implant with force fit between implant and bone (d).

**Figure 10:**
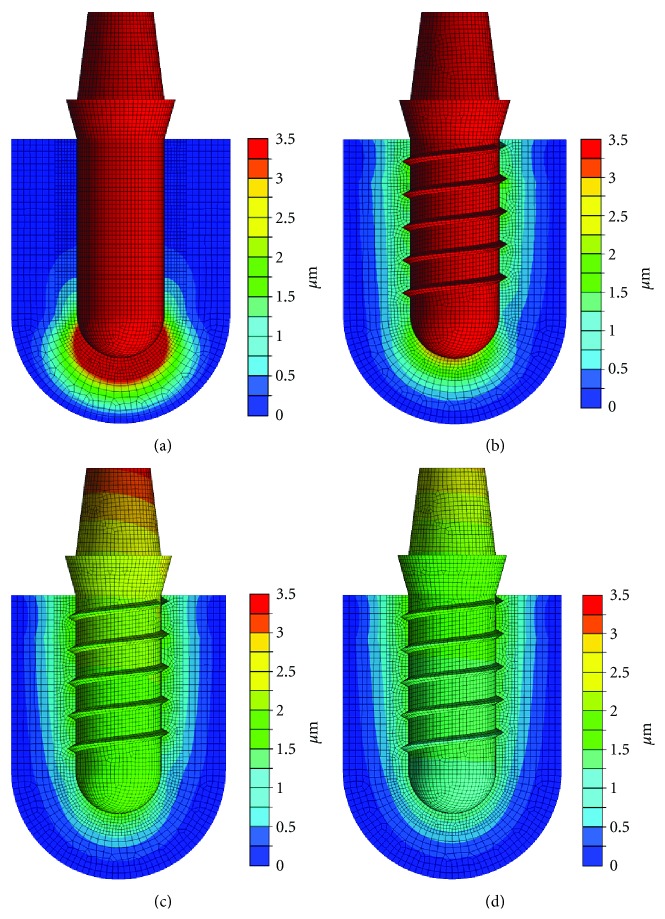
Distribution of global displacement around implants loaded with 200 N axial vertical force [12]: cylindrical implant without friction between implant and bone (a), threaded implant without friction between implant and bone (b), threaded implant with friction between implant and bone (coefficient of friction: 0.3) (c), and threaded implant with force fit between implant and bone (d).

Simulating 200 N axial force acting on an osseointegrated cylindrical implant with no friction between implant and bone caused a symmetric loading situation of the bone surrounding the implant with maximum loading and maximum displacement occurring at the apical part of the implant (Figures 9(a) and 10(a)).

Adding threads to the implant led to a decrease both in loading and displacement occurring at the apical part of the implant. Simultaneously, greater distribution of loading and displacement was observed at the vertical walls of the implants (Figures 9(b) and 10(b)).

Introducing friction between implant and bone (Figures 9(c) and 10(c)) further decreased global displacement and resulted in a more homogeneous distribution of loads as compared to the force fit situation (Figures 9(d) and 10(d)), where load transfer predominantly occurred in the cervical area of the implant, where cortical bone was modelled.

**Figure 11:**
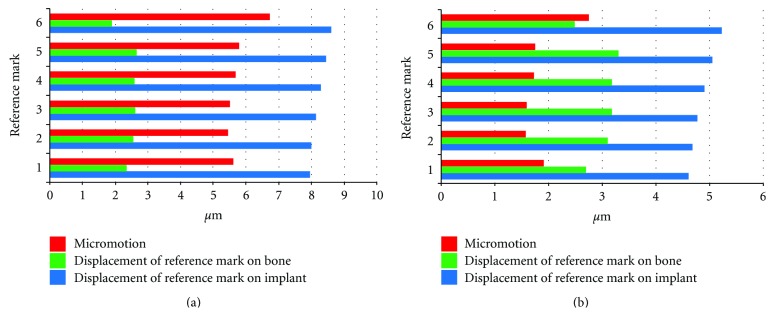
Displacement of corresponding reference marks on bone and implant for freshly inserted implants and resulting micromotion: data recorded from model without friction between bone and implant (a), data recorded from model with friction between bone and implant (b) (note the different scales).

For freshly inserted implants with a soft intermediate layer of bone modelled around the implants, the introduction of a friction coefficient led to a considerable reduction in micromotion between implant and bone as well as to reduced displacement of all reference marks on the implant. Displacement of the reference marks on the bone remained on a constant level. Overall, comparable values for micromotion were recorded at all corresponding reference marks (Figure 11).

**Figure 12:**
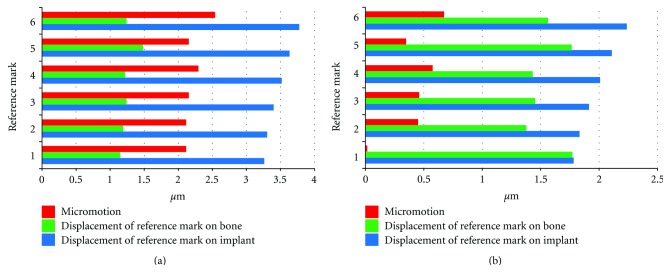
Displacement of corresponding reference marks on bone and implant for osseointegrated implants and resulting micromotion: data recorded from model without friction between bone and implant (a), data recorded from model with friction between bone and implant (b), note the different scales!

Simulating an osseointegrated implant in general reduced all displacement values by about 50% compared to the situation of a freshly inserted implant. Again the introduction of a friction coefficient led to a considerable reduction in micromotion between implant and bone as well as to reduced displacement of all reference marks on the implant. Displacement of the reference marks on the bone remained on a constant level. In contrast to a freshly inserted implant, the distribution of micromotion depended on the location of the reference mark. Whereas in the cervical aspect some minor micromotion in the range of 0.75 *μ*m could be found, at the most apical reference almost no relative displacement between implant and bone occurred (Figure 12).

## 4. Discussion

Within the limitations of this investigation, the effect of friction phenomena and implant design (cylindrical versus threaded) on stress distribution and implant displacement could be demonstrated. Both the introduction of friction between implant and bone as well as the addition of threads to a cylindrically shaped implant resulted in the reduction of implant displacement under an axial load of 200 N. Simultaneously, a more homogeneously distributed loading situation at the implant bone interface could be observed. Changing the contact type between implant and bone to force fit resulted in load transfer predominantly occurring in the cervical part of the implant surrounded by stiffer cortical bone. This is in strict contrast to a situation with no friction modelled resulting in maximum loading of bone surrounding the periapical region of the implant. From a clinical perspective, these findings indicate that screw-shaped implants are advantageous while bone quality probably plays the most important role in achieving sufficient primary implant stability for immediate loading. All these factors should be taken into account when choosing a specific loading protocol.

Based on a comparison of freshly inserted and osseointegrated implants it could be shown that the healing status affects the occurrence of micromotion phenomena along the implant bone interface. For a soft implant bone interface, reflecting early stages of osseointegration, micromotion remained on a constant level regardless of the location considered. Simulating mature bone reflecting an osseointegrated implant, the introduction of a friction coefficient between implant and bone dramatically changed the distribution of micromotion along the implant bone interface. In addition to generally reduced levels of micromotion as compared to a freshly inserted implant, a decrease in micromotion was noted. The amount of micromotion decreased towards the apex of the implant.

It may be seen as a limitation of this study that only one specific value for axial loading of the implants was chosen. Based on studies by Brunski and coworkers [20], axial components of biting forces can range from 100 to 2400 N, while the exact values depend on factors such as location in the mouth and nature of food. For patients having implant-supported dentures, axial closure forces ranging from 45 to 255 N have been reported [25]. It thus appears that the value chosen reflects clinical loading magnitudes.

Furthermore, besides the pure mechanical aspects addressed in this paper, also biologic factors play an important role in the process of osseointegration of dental implants. Following implant placement, the healing period starts with the adherence of serum proteins, followed by the attachment and proliferation of mesenchymal cells. Consequently, osteoid is formed in what is then mineralized. From then onwards, bone remodeling occurs as an adaptation to the implants environment [26]. With these processes occurring simultaneously to mechanical loading in an immediate loading situation, the interaction of both mechanical and biologic factors seems to be critical to the integration of the implant. 

## 5. Conclusions

Given the nonuniform distribution of micromotion between implant and bone, it appears questionable whether currently available methods for experimentally determining this phenomenon provide meaningful data. The only valid approach for evaluating micromotion phenomena at the implant-bone interface appears to be finite element analysis. However, care has to be taken to set proper materials and interface characteristics as these parameters may greatly influence the outcome.

## Figures and Tables

**Table 1:** Material properties (Young's moduli in MPa) chosen in the different models. Poisson's ratio is 0.3 for all materials.

Structure	Osseointegrated implant	Healing state
Cortical bone	14000	14000
Trabecular bone	3000	3000
Implant	110000	110000
Intermediate layer—cortical area	14000	1000
Intermediate layer—trabecular area	3000	1000
